# Feature sensitivity criterion-based sampling strategy from the Optimization based on Phylogram Analysis (Fs-OPA) and Cox regression applied to mental disorder datasets

**DOI:** 10.1371/journal.pone.0235147

**Published:** 2020-07-01

**Authors:** Fatemeh Gholi Zadeh Kharrat, Newton Shydeo Brandão Miyoshi, Juliana Cobre, João Mazzoncini De Azevedo-Marques, Paulo Mazzoncini de Azevedo-Marques, Alexandre Cláudio Botazzo Delbem

**Affiliations:** 1 Department of Bioengineering, Universidade de Sao Paulo Escola de Engenharia de Sao Carlos, Sao Carlos, Sao Paulo, Brazil; 2 Center of Information and Informatics of Medical School, Ribeirao Preto, Universidade de Sao Paulo Escola de Enfermagem de Ribeirao Preto, Sao Paulo, Brazil; 3 Department of Mathematics and Statistics, Universidade de Sao Paulo Instituto de Ciencias Matematicas e de Computacao, Sao Carlos, Sao Paulo, Brazil; 4 Department of Social Medicine of Medical School, Universidade de Sao Paulo Campus de Ribeirao Preto, Ribeirao Preto, Sao Paulo, Brazil; 5 Department of Medical Imaging, Hematology and Clinical Oncology of Medical School, Universidade de Sao Paulo Campus de Ribeirao Preto, Ribeirao Preto, Sao Paulo, Brazil; 6 Department of Computer Science, Universidade de Sao Paulo Instituto de Ciencias Matematicas e de Computacao, Sao Carlos, Sao Paulo, Brazil; National Institutes of Health, UNITED STATES

## Abstract

Digital datasets in several health care facilities, as hospitals and prehospital services, accumulated data from thousands of patients for more than a decade. In general, there is no local team with enough experts with the required different skills capable of analyzing them in entirety. The integration of those abilities usually demands a relatively long-period and is cost. Considering that scenario, this paper proposes a new Feature Sensitivity technique that can automatically deal with a large dataset. It uses a criterion-based sampling strategy from the Optimization based on Phylogram Analysis. Called FS-opa, the new approach seems proper for dealing with any types of raw data from health centers and manipulate their entire datasets. Besides, FS-opa can find the principal features for the construction of inference models without depending on expert knowledge of the problem domain. The selected features can be combined with usual statistical or machine learning methods to perform predictions. The new method can mine entire datasets from scratch. FS-opa was evaluated using a relatively large dataset from electronic health records of mental disorder prehospital services in Brazil. Cox’s approach was integrated to FS-opa to generate survival analysis models related to the length of stay (LOS) in hospitals, assuming that it is a relevant aspect that can benefit estimates of the efficiency of hospitals and the quality of patient treatments. Since FS-opa can work with raw datasets, no knowledge from the problem domain was used to obtain the preliminary prediction models found. Results show that FS-opa succeeded in performing a feature sensitivity analysis using only the raw data available. In this way, FS-opa can find the principal features without bias of an inference model, since the proposed method does not use it. Moreover, the experiments show that FS-opa can provide models with a useful trade-off according to their representativeness and parsimony. It can benefit further analyses by experts since they can focus on aspects that benefit problem modeling.

## Introduction

In the last decades, thousands of patients have their data storage into digital datasets. Nevertheless, those datasets have not been analyzed in a way that all the possible relationships among all the data are verified. In general, it would require several experts in different types of knowledge working in an integrated way. The lack of available professionals for such work usually involves the problem domain (e.g., health, energy, finance, agribusiness, etc.) and/or data science areas (statistics, artificial intelligence, optimization, high-performance computing, etc.). Emphasis on innovations to deal with large-scale dataset has increased recently, in some forms called BigData [1], it has motivated the development of new computing methods. In the healthcare domain, this challenge is even greater, since it may involve a lot of distinct knowledge from experts, making hard to develop automated analysis.

On the other hand, *Estimation of Distribution Algorithms (EDAs)* composes an area of investigation of optimization strategies that aim at learning a problem from scratch to create models for search space exploration [2] Although the efficacy verified for some of them, they showed not properly for several large-scale real-world problems, in fact, the majority of the success cases are related to some problem categories. Recently, a new *EDA*, called *Optimization based on Phylogram Analysis) OPA (*[3] overcame those drawbacks, obtaining relevant results for different data types, as well as mixed data types. Moreover, *OPA* scalability was proved for binary problems [4] and it has been experimentally verified for some real-world problems.

Such new scenario open opportunities to extend those results for data mining of complex datasets. This paper presents an *OPA* extension to construct a problem model from raw data. The first version of the model consists in selecting the main variables of features from the dataset. It means to find a set of consistent variables, those with enough information, but without repetition, among other aspects. Note that variable correlation (related to common information among variables) is one of the main aspects learned by *EDAs* to construct probabilistic models.

The challenges in this context of healthcare datasets can also involve other data aspects, beyond size and their data meaning. Usually the factors used for performing analysis by experts motivated the procedures for data acquisition, the data structure of the storage, among other aspects that make them biased by demands that were relevant (a) decade(s) ago. The dynamics of population and technology can make them asynchronous to current demands, which can generate data inconsistency. Such an issue is hard to automate for large datasets. A typical concern is time-space granularity and its consistency with an analysis goal. Another critical aspect of health data is the constrained access to information due to its sensitiveness for patients, which is more complex when considering the whole dataset from large health centers. Fortunately, hospitals and prehospital facilities for mental health disorder in the Ribeirao Preto region in Brazil succeed in generated a public and reliable dataset, as shows the achievements of Barros et al [5] and Miyoshi et al. [6] Living with mental health problems are a serious personal and social challenge that has a profound impact, not only on patients, their family, and the health services but also on the economy, which is given that mental health disorders are the most costly condition in low-income and middle-income countries [7–9] Whereas the recognition of mental disorders has risen over the last decades, the Length of Stay (LOS) in psychiatric hospitals has increased. So, understanding factors associated with the indicator of LOS for mental health disorders not only can be critical to managing the quality of care and economic reasons but also, insurers, administrators and policymakers are interested in the predictors of the length of each hospitalization [10,11]. Some previous studies have determined that different factors tend to effect on the LOS. In general, they found heterogeneous conclusions. For instance, some of them suggested the demographic variables such as age, gender, marital status, type of admission, place of residence and employment status [7,12–15] On the other hand, other aspects as clinical variables, administrative information [16–18], suicide attempts and homelessness [19] are reported as the most important. Until a certain point, the heterogeneous conclusions also illustrate the discorrelation that may take place involving the types of features stored and a purpose of an analysis, which can increase with the dynamics of population and technology.

Such new scenario open opportunities for investigations of new data mining approaches, as the proposed here based on *OPA*. When constructing a problem model from raw data, *OPA* can automate a series of data mining procedures, without the bias for an analysis goal. In fact, *OPA* generates several models that evolve according to general criteria, related to representativeness and parsimony. The first step of modeling consists of selecting the main variables (features) from the dataset. It produces a set of consistent variables, that should be small but with enough information according to the criteria, without redundancy, among other aspects. Note that variable correlation (related to common information among variables) is one of the main aspects learned by *EDAs* to construct probabilistic models.

In order to deal with a variety of data types in the same dataset as well as the absence of domain knowledge for pre-processing data, *DAMICORE* [20] method is used for the model construction. Basically, it finds variable correlations and represents them in a graph tree (a raw model). Based on such results, some feature sensitivity was proposed, composing a new method that we called Feature Sensitivity based on *OPA* (*FS-opa*).

The following objectives can synthesize the investigation we carried on: 1- Development of a method that can directly work with complete raw datasets; 2- Verification of its capabilities using a real-world datasets and the relevance of information extracted from data; 3- A model that can benefit further analysis by experts, and 4- the development of a *FS-opa* procedure to enable the inclusion of problem domain knowledge. Note that selecting the principal features is a way to obtain parsimonious inference models, which can make easier for further analysis by experts as well as it may reduce collinearity and eventually improve the accuracy of predictors.

The remaining of the paper is organized as follows. First presents the main aspects of the dataset used and proposes *FS-opa*. The second shows the experiments with *FS-opa* for the mental disorder dataset. After that presents results and discussion of Cox model, combine with *FS-opa*. Finally, concludes the paper.

## Material and methods

The dataset applied and the main computer methods investigated for developing the proposal are arranged in the sequel. Mental disorder dataset section describes some aspects of the mental disorder dataset studied. Estimation of Distribution Algorithms (EDAs) section presents the Optimization based on Phylogram Analysis, which possesses some properties that are relevant for working with raw data in bases of relatively large amount of features.

### Mental disorder dataset

The investigation here presented uses the dataset of mental health care collected by the information system of the Coordination of Hospitalizations in Ribeirao Preto, Brazil, from July 2012 to December 2017 [6,21]. The dataset contains information on 8,755 patients with an average age of 37.6 years. The features of the dataset are related to: 1- socio-demographic aspects, 2- information for people admission in a hospital stay (location, duration, patient origin and destination), 3- diagnoses (at admission, primary diagnosis and secondary diagnosis), 4- services used (such as transfer patients to other hospitals), 5- Information on inpatient discharges from hospitals that provide general or specialized care, 6- date information such as (date of registration, date of discharge and date of death, etc.), 7- codes associated to patients, hospitals and hospitalization procedures, 8- mental diagnostic codes (according to the International Classification of Diseases, 10th Revision—ICD-10).

Next we highlight some groups of those disease presented on the dataset: neurotic and anxiety disorders (F40-F49), psychotic disorders including schizophrenia (F20-F29), bipolar disorder (F30, F31), depressive disorders (F32-F34), personality disorders (F60-F69), alcohol-related disorders (F10), other substance-related disorders (F11-F19), and other mental disorders (mainly F00-F09 and F70-79). Moreover, the records include information about other types of diseases, such as a heart problem, trauma, and stroke. All the fifty-two Portuguese and English names of all the features (also denoted here as variables) are shown in S1 Appendix. The corresponding dataset is referred as both 52-feature dataset (52-FD) and raw-and-Full dataset (raw-Full FD), according to the purpose.

### Estimation of distribution algorithms (EDAs)

**Fig 1:**
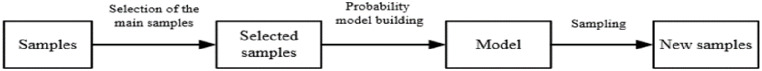
Overview of EDAs.

*EDAs* are optimization methods based on evolutionary theory that automatically constructs a problem model, which is used to search on the decision space. They require relatively few parameters to setup. Basically, an *EDA* uses samples of variable values from promising solutions of a problem to generate a model of variable correlations and probabilities. Then, new values for each variable (a candidate solution) are generated from the model, sampling the decision space. After selecting the best-found solutions among those new generated, a new model can be produced and start a new cycle of the *EAD* processing. (Fig 1) synthesizes the main steps of general *EDAs*.

The main drawbacks of them have being the computing time to construct the models, since high quality models (that can properly represent a problem) usually requires significantly more computation. For example, Bayesian Networks [22] can construct models with high quality for several complex problems, but the corresponding running time can make the complete processing unfeasible for large instances of them (high number of variables, large groups of correlated variables, mixed data type, etc.).

## Optimization based on phylogram analysis

**Fig 2:**
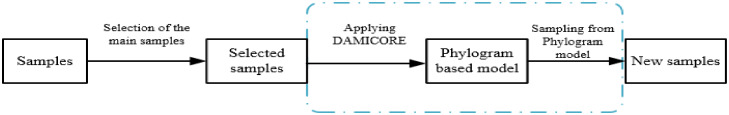
Optimization based on Phylogram Analysis-OPA.

*OPA* [3] is an *EDA* that can guarantee an adequate tradeoff between computing time and the quality of problem models for some complex problems. Models of *OPA* are phylograms (as the phylogenetic trees used to describe species evolution) combined with joint probabilities of variables associated to each phylogram subtree (clades). Relatively fast algorithms can construct useful phylograms. *OPA* uses Fast Algorithm of Newman to generate models from large amount of data (that can also be of any type) and a resampling technique to overcome bias from greedy procedures involved in this algorithm and from data samples. Those characteristics make *OPA* proper for dealing with new data sets and problem domains with poor knowledge available (where there is no previous model or expert to orient a modeling process). (Fig 2) shows a flowchart of the *OPA* main steps. The optimization cycle of an *OPA* is like a typical *EDA*, differing mainly by the method of model construction.

Next, we propose an approach, called Feature Sensitivity through criterion-based resampling from OPA (*FS-OPA*), based on *OPA* to identify the principal features (variables) of a problem from scratch. Thus, problem models can be generated for any problem if some significant amount of data is available. This type of result can reduce the time of the investigation until one reaches a useful result or preliminary conclusions, benefiting analysis for different fields, mainly the ones with lack of funds or expert practitioners to carry on the investigations.

A Feature Sensitivity analysis aims at finding a set with the principal features of the problem taking into account both the current context (e.g., sampled data quality and its relevance for a purpose) and the feature interactions [23]), which differs from usual feature selection approaches. *FS-OPA* can perform the feature sensitivity analysis for any type of data and grade of interactions. The results are arranged in a model that highlights feature relationships and relevance (based on phylogram(s)). The main values of variables relevant for the success are represented by joint probabilities, as in *OPA*, conditional probabilities, a regression method, classification approach, etc. *FS-OPA* was hybridized to a Cox approach to enable survival analysis. Then, the output of *FS-OPA* is a probabilistic model. The synthesis of a system in such model enables practitioners to realize mechanisms of relatively complex systems, as well as to make decisions with higher level of confidence. Next Section introduces *FS-OPA*.

### Feature sensitivity through criterion-based resampling form OPA

**Fig 3:**
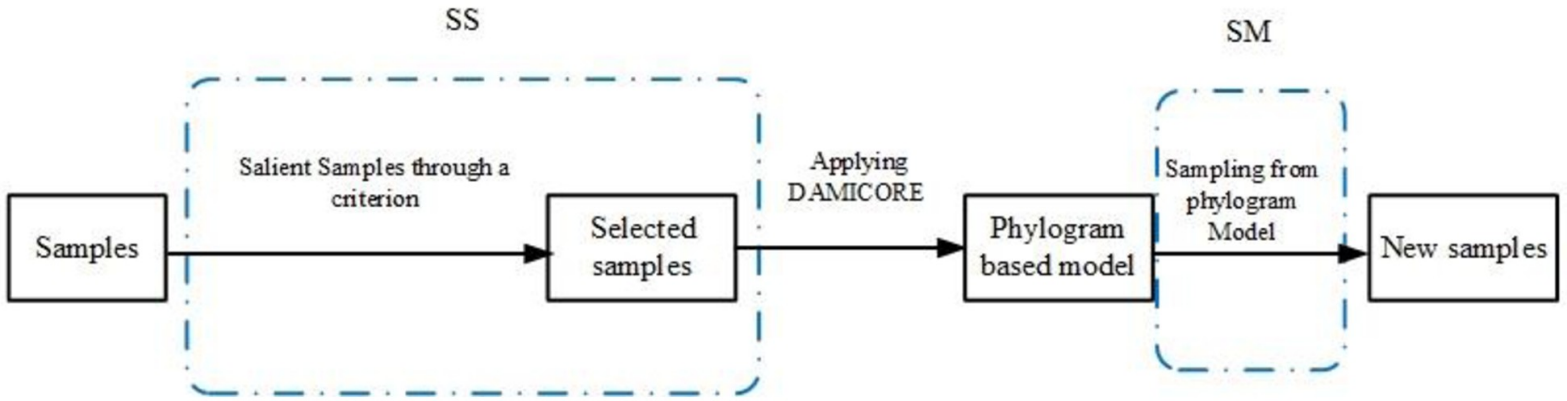
SS and SM rectangles in blue dashed lines highlight the OPA steps modified for the construction of FS-OPA.

FS-OPA is described from by the modifications of it highlighted in (Fig 3). They SS (Salient Samples through a criterion) and SM (Sampling from a phylogram Model). Next Sections present both SS and SM.

### SS in FS-OPA

Tournament operator is a procedure used by *EDAs* to highlight promising regions in the search space. *OPA* performs it through selecting points (solutions/samples) in such regions, according to a purpose or optimization criterion. It picks up randomly a small number of samples (as individuals that compete in a tournament) and saves the best of them according to the criterion (also called objective function or fitness) in a set. This process repeats until the set has enough samples for modeling. In a certain way, the tournament is a resampling method with similar benefits, as it reduces the data unbalance bias, among other aspects that benefit the modeling process. The selection pressure (usually two, denoted as *s* = 2) is the number of individuals that compete in each tournament and it is a parameter to setup. Another relevant operator used by *EDAs* to salient promising regions of the search space is called ranking. After sorting all samples (according to a criterion) into a vector, the first best segment [8] of the vector composes the set of selected samples. The size of the top is adjustable by the selection pressure imposed (size of the top is equal to equal to $\frac{n}{s}$ where n is the total amount of number of samples), managed as a parameter.

*FS-opa* bypasses such a parameter by developing a selection that works with three levels of selection pressure. It uses ranking selection for the sake of simplicity in the code implementation and validation since it is a deterministic procedure. Besides, the stochasticity added by the use of the three levels can contribute to the reduction of bias. First, *FS-opa* ranks all the dataset according to the used criterion (e.g., minimizing LOS), generating an ordered sequence, a rank R. The three levels of selection pressures result in three levels of categorizations of R. First, FS-opa ranks all the dataset according to the used criterion (e.g., minimizing LOS), generating an ordered sequence, a rank R. The three levels of selection pressures result in three levels of categorizations of R. Differently from *OPA*, FS-opa saves both the samples at the top (head of the rank) and the bottom (tail of the rank) of each categorization:1- two-level categorization (2*C*, *s* = 2), 2-four-level categorization (4*C*, *s* = 4), and 3- eight level categorization (8*C*, *s* = 8).

**Fig 4:**
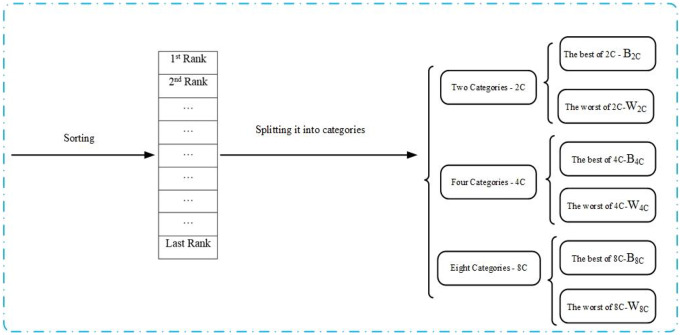
Components of SS in FS-opa that find relevant sample subsets B_2C_, W_2C_, B_4C_, B_8C_, W_4C_, and W_8C_.

A set called *B*_2*C*_ saves the best samples (according to the criterion) from 2*C*, and another, *W*_2*C*_ stores the worst samples from it. Similarly, *B*_4*C*_ (*B*_8*C*_) is the head with best samples after splitting R into four (eight) categories, and *W*_4*C*_ (*W*_8*C*_) is the tail with the worst samples. (Fig 4) summarizes the SS procedure. Note that highest pressure can increase the number of categorizes subsets, however, results based on *OPA* show that those three values are usually enough for variety of complex problems [3].

### SM in FS-opa

**Fig 5:**
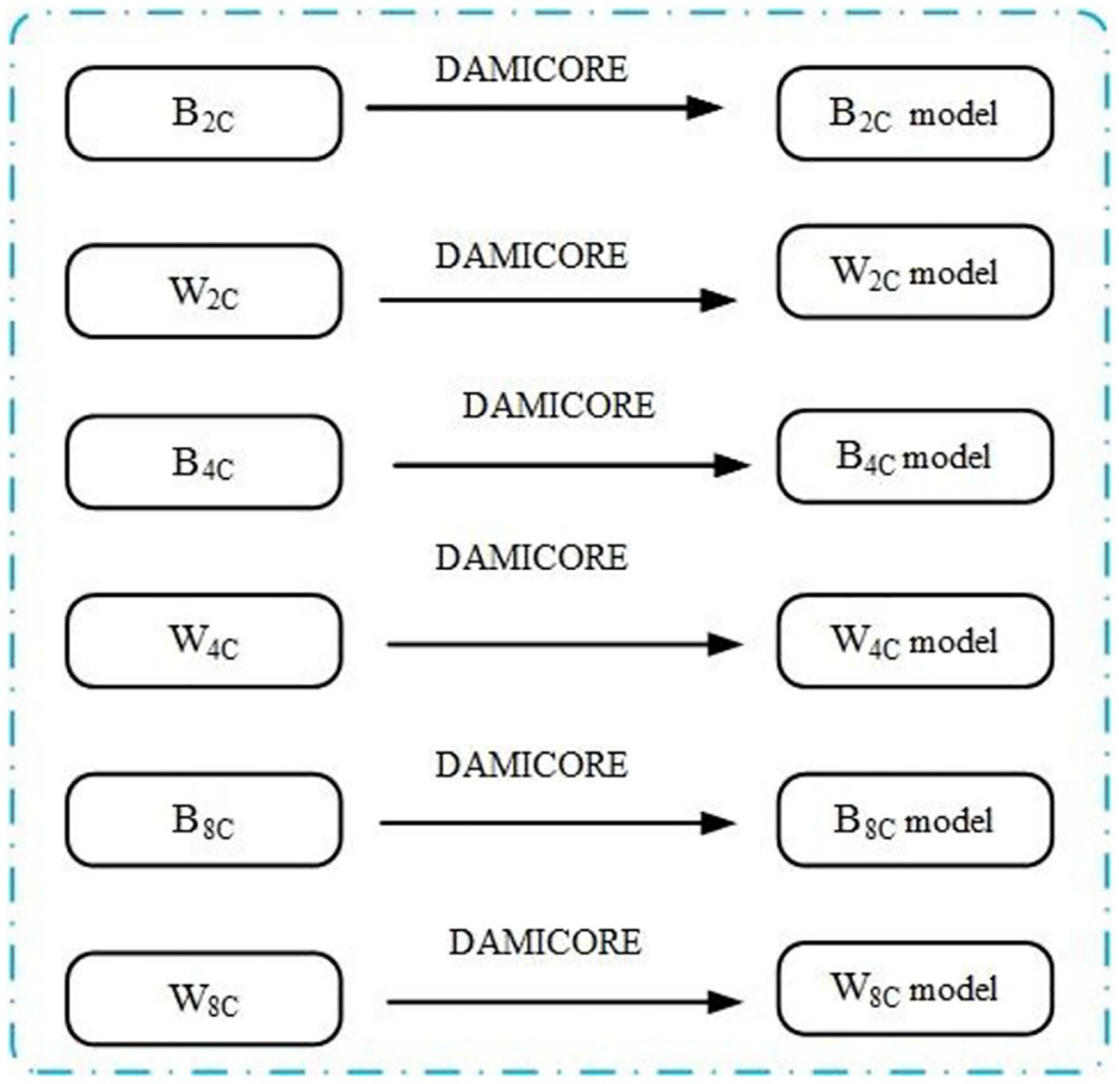
SM constructs six phylogram-based models.

The execution of the model-building strategy of *OPA* for each of the six subsets generated by SS produces six phylogram-based models. (Fig 5) synthesizes the SM procedure. The phylograms represent the main relationships among variables (also called features or factors, depending on the domain). Based on them, we determine a set of principal features, as described in the sequel.

The algorithm used by SM for phylogram constructions is *DAMICORE* [3]. This method can work with data from any type and structure (integer, real and complex numbers, categorical data, images, sound, movies, etc.), as well as mixed data types, without any pre-processing (filtering, outlier detection, feature extraction, among others). *DAMICORE* associates to each phylogram possible clusters of the strongly correlated objects in the analysis, where each object corresponds to a vector with the values of a variable manipulated by *FS-opa*. Moreover, it requires no parameter set up to run, although another code compressor (gzip is the default choice) improving compression can benefit the resulting quality.

**Fig 6:**
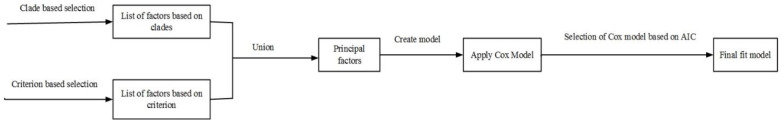
Feature sensitivity from models and their use in the Cox approach.

SM applies DAMICORE to the six subsets (*B*_2*C*_, *W*_2*C*_, *B*_4*C*_, *W*_4*C*_, *B*_8*C*_, *W*_8*C*_ samples (Fig 6), but the objects for *DAMICORE* manipulation are values associated to each variable. If data has a spreadsheet structure, each entire column corresponding to a variable (*x*_*i*_) composes an object (also called *x*_*i*_). In practice, a file called $x_{i}^{B_{2C}}\;(x_{i}^{W_{2C}},\;x_{i}^{B_{4C}},x_{i}^{W_{4C}},x_{i}^{B_{8C}},\;x_{i}^{W_{8C}}$), saved in a directory/folder called *B*_2*C*_ (*W*_2*C*_, *B*_4*C*_, *W*_4*C*_, *B*_8*C*_, *W*_8*C*_), stores the data associated with variable *x*_*i*_ from samples in *B*_2*C*_ (*W*_2*C*_, *B*_4*C*_, *W*_4*C*_, *B*_8*C*_, *W*_8*C*_). It results in six directories, each one with *l* variables, where *l* is the total amount of variables of the problem (number of columns of a spreadsheet). The size of files $x_{i}^{B_{2C}}$, and $x_{i}^{W_{8C}}$, are $\frac{n}{2}\;$ in *B*_2*C*_ and *W*_2*C*_, respectively. Similarly, the sizes of files $x_{i}^{B_{4C}}$ and $x_{i}^{W_{4C}}\;(x_{i}^{B_{8C}}$ and $\;x_{i}^{W_{8C}})$ are $\frac{n}{4}\;(\frac{n}{8})$. Note that the size of files (number of samples used) decreases as the selection pressure increases. Finally, *DAMICORE* runs for each directory generating the six phylograms (preliminary models) with clusters associated (also called clades in the domain of evolution theory that used here by convenience).

(Fig 6) illustrates the analysis of the feature sensitivity of SM, organized into two techniques: one strictly based on clades found for each of the six categorized subsets (*B*_2*C*_, *W*_2*C*_, *B*_4*C*_, *W*_4*C*_, *B*_8*C*_, *W*_8*C*_) and another based on the sizes of the graph paths from a reference variable (leaf node) to the other variables (also leaf nodes) calculated for each phylogram. The former process is called SM1 and the latter, SM2. Note that finding the principal and less correlated factors is a way to find a parsimonious inference model. They can benefit further analysis by experts since they can focus on the more relevant aspects of a relatively complex system. Moreover, fewer features may reduce collinearity and improve the accuracy of prediction models until a certain point.

First, SM1 splits phylograms into two clades: *C*_1_ and *C*_2_. Supposedly, the majority of the objects in each clade have low-level common information. Thus, the remotion of the branch (graph edge) in the middle of the largest (graph) path of the phylogram generates such clades. Note that other strategies to estimate the largest uncorrelated clades from a phylogram are possible according to *OPA* principles, but the middle-path-based criterion is relatively simple and enough to produce relevant results. Moreover, if the largest path has an even number of branches, both remotions are tested and the one with higher congruence in the procedure described in the next paragraph is chosen.

SM1 applied to the six preliminary models produces twelve clades, denoted: *C*_1_*B*_2*C*_, *C*_2_*B*_2*C*_, *C*_1_*W*_2*C*_, *C*_2_*W*_2*C*_, *C*_1_*B*_4*C*_, *C*_2_*B*_4*C*_, *C*_1_*W*_4*C*_, *C*_2_*W*_4*C*_, *C*_1_*B*_8*C*_, *C*_2_*B*_8*C*_, *C*_1_*W*_8*C*_, *C*_2_*W*_8*C*_. Then, SM1 compares nodes from clade *C*_1_*B*_2*C*_ (*C*_1_*B*_4*C*,_ *C*_1_*B*_8*C*_) with both the nodes from *C*_1_*W*_2*C*_ (*C*_1_*W*_4*C*_, *C*_1_*w*_8*C*_) and *C*_2_*W*_2*C*_ (*C*_2_*W*_4*C*_, *C*_2_*W*_8*C*_) to find the most congruent clade pair (those with the most nodes in common, suppose it is (*C*_1_*B*_*iC*_, *C*_1_*W*_*iC*_)). This pair and the remaining clades in each category compose respectively pairs ${p\;}_{1}^{i}\;$ and ${p\;}_{2}^{i}$, for each category *i* (*i* in {2*C*, 4*C*, 8*C*}). In other words, ${p\;}_{1}^{i}\;=\;(C_{1}B_{iC},\;C_{1}W_{iC})$ and ${\;p\;}_{2}^{i}\;=\;(C_{2}B_{iC},\;C_{2}W_{iC})$ assuming the most congruent pair is (*C*_1_*B*_*iC*_, *C*_1_*W*_*iC*_); otherwise, ${p\;}_{1}^{i}\;=\;(C_{1}B_{iC},\;C_{2}W_{iC})$ and ${\;p\;}_{2}^{i}\;=\;(C_{2}B_{iC},\;C_{1}W_{iC})$ is the most congruent pair.

Suppose ${p\;}_{1}^{i}$ is (*C*_1_*B*_*iC*_, *C*_1_*W*_*iC*_). SM1 selects the leaf nodes whose siblings changed in the corresponding phylograms when comparing any pair (*x*, *y*) of leaf nodes from ${p\;}_{1}^{i}$, *x* in *C*_1_*B*_*iC*_ and *y* in *C*_1_*W*_*iC*_. Then, SM1 stores the leaf nodes (selected features) from ${p\;}_{1}^{i}$ into ${s\;}_{1}^{i}$. SM1 runs also for ${p\;}_{2}^{i}$ producing $\;{s\;}_{2}^{i}$. The output of SM1 is called a clade-based list, *s*_*clade*_, corresponding to the union of ${s\;}_{1}^{i}$ and ${s\;}_{2}^{i}$ for all *i*.

**Fig 7:**
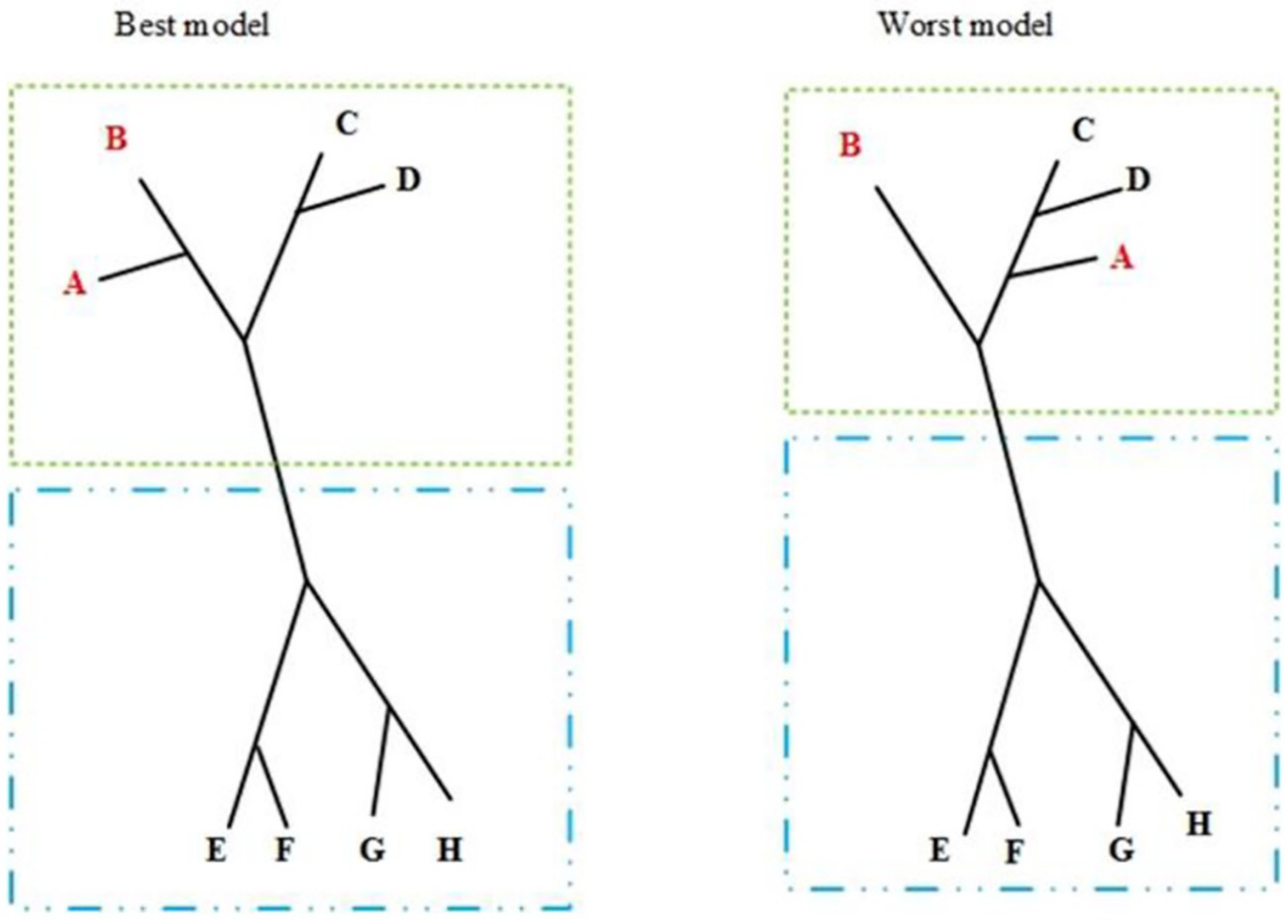
Clade-based list: ${p\;}_{1}^{i}\;({p\;}_{2}^{i})$ with clades rounded by green (blue) dashed lines. ${s\;}_{1}^{i}\;=\;\{A,B\}$ and ${s\;}_{2}^{i}\;=\;\{\}$, therefore, *s_clade_* = {*A*, *B*}.

Note that features (leaf nodes) whose siblings (relationships) changed from the model based on the best samples to the model constructed from the worst samples should have enough information to identify each of these two types of samples. Thus, those features are expected to be meaningful for problem modeling and solving.

(Fig 7) synthesizes the clade-based sensitivity by SM1 from two phylogram models, highlighting clades *C*_1_ and *C*_2_, respectively, from the best and the worst models. ${p\;}_{1}^{i}$ is pair (*C*_1_*B*_*iC*_, *C*_1_*W*_*iC*_), where *C*_1_*B*_*iC*_ and *C*_1_*W*_*iC*_ are the clades surrounded by the green dashed lines respectively in the best and worst models. Similarly, ${p\;}_{2}^{i}$ is pair (*C*_2_*B*_*iC*_, *C*_2_*W*_*iC*_) with clades highlighted by dashed blue lines. The siblings changed are in ${s\;}_{1}^{i}\;=\;\{A,B\}$ (the leaf nodes in red color) and ${s\;}_{2}^{i}\;=\;\{\}$, thus, *s*_*clade*_ = {*A*, *B*}.

**Fig 8:**
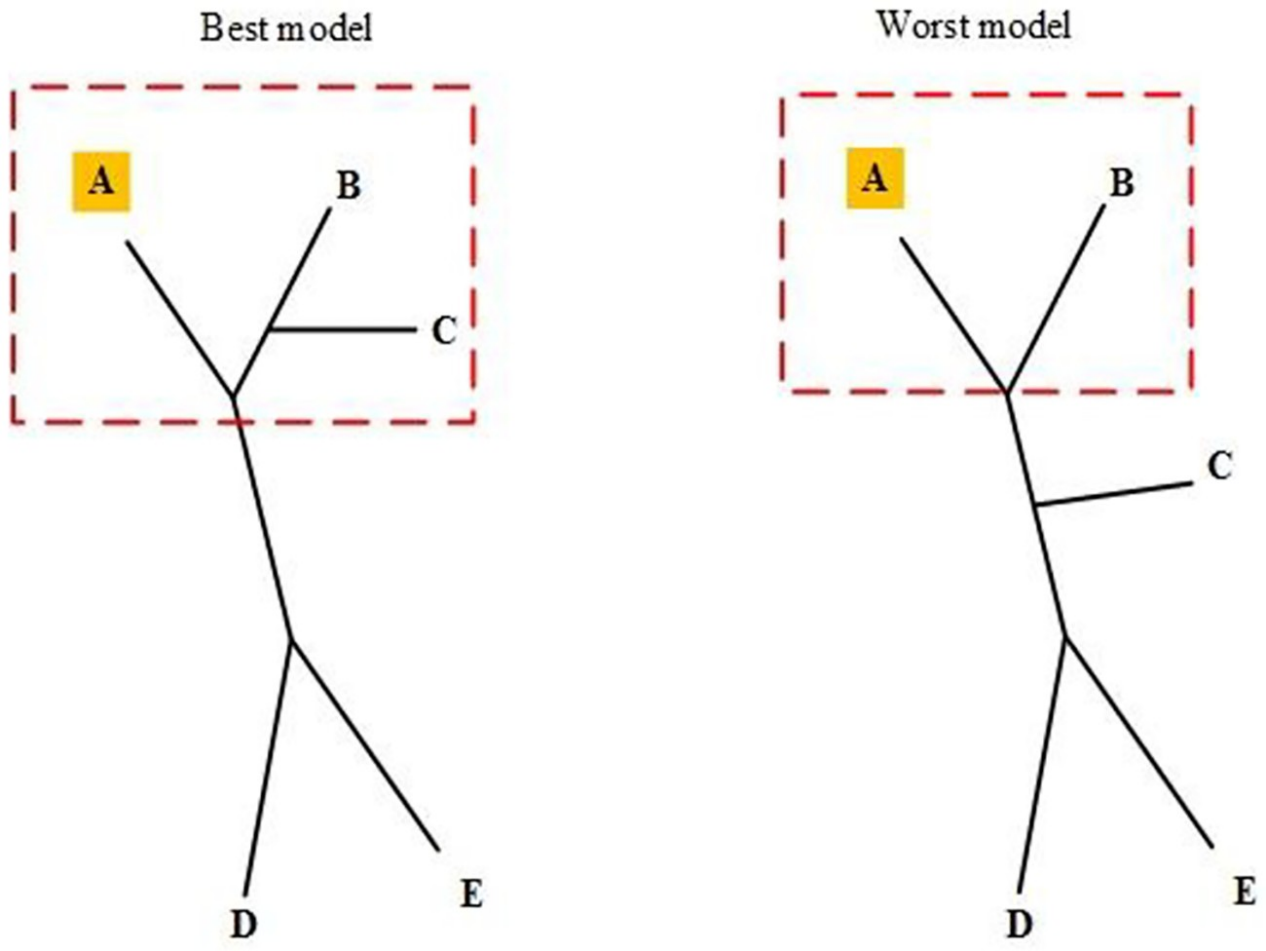
Criterion-based lists, Red dashed lines highlight subtrees found by SM2 using node A as a target feature. s_criterion_ = {A, B, C}.

SM2 uses the same four clades found by SM1 (*C*_1_*B*_*iC*_, *C*_2_*B*_*iC*_, *C*_1_*W*_*iC*_, *C*_2_*W*_*iC*_) for each category *i*. It also uses a unique pair ${q\;}_{1}^{i}$ composed by the clades from the best and the worst models that have the target feature (leaf mode).

(Fig 8) highlights a target, feature *A* (yellow), and the clades of ${q\;}_{1}^{i}$ (red-dashed lines) with it. The target is a feature with strong relation to a goal according to a criterion or a purpose of the analysis from the problem model. Such feature is called the target criterion.

Fig 8 also shows the two clades of pair ${\;q\;}_{1}^{i}$. The clade at left has two leaf nodes, *A* and *B*, in the subtree whose root is the sibling of the target feature, *A*. The clade at right has the sibling of *A* corresponding to leaf node *B*. Thus, $s_{criterion}^{i}$ results in {*A*, *B*, *C*}.

Note that SM2 requires some previous knowledge from the problem domain. Since it can indicate relevant features, SM2 runs based on them to compose *s*_*criterion*_. This property of SM2 seems useful for adaptive approaches with human intervention when, for example, practitioners motivated by some partial results manipulate the dataset or the found models according to their experience. In this way, *FS-opa* can deal with several levels of knowledge from the problem domain, benefiting from it, when possible. For example, in our dataset, other features have some relation to LOS. Each of those features can also be treated as a target to improve problem modeling. In such a case, SM2 should run for each of the new targets generating new criterion-based lists that must be combined through the union operator.

Finally, the lists produced by SM1 and SM2 are combined in a same set *r*, by applying the union operator to *s*_*clade*_ and *s*_*criterion*_. Based on *r*, regression methods, machine learning, among other approaches, can construct complete models for prediction, diagnosis, etc.

### Cox approach in FS-opa

**Fig 9:**
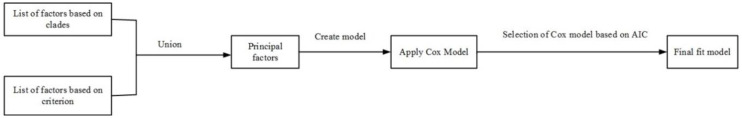
Feature sensitivity from models and their use as the main variables in the Cox approach.

Our purpose of investigation in this project concerns on time length of treatment or staying in hospitals, then survival analysis is a relevant strategy to construct a stochastic problem model. Cox approach is used to complete a stochastic phylogram-based modeling through *FS-opa*. (Fig 9) illustrates the integration of SM and Cox approach in *FS-opa*.

Cox regression model is a semi-parametric regression model, which is the most popular in common in medical research to analysis data with time to discharge [24–26]. For each patient *i* in the dataset, we have the times of hospitalization *m*_*i*_, *m*_*i*_ is the number of observed events for patient *i*. Thus, the LOS for the *i*^*th*^ patient and for *j*^*th*^ stay is given by *t*_*ij*_ − *t*_*ij*−1_ where *T*_*i*,0_ = 0, for all *i* = 1, …, *n* and *j* = 1, …, *m*_*i*_, where *n* is the total number of patients. The hazard function for the *j*^*th*^ event of *i*^*th*^ subject at time *t*, *i* = 1, …, *n* and *j* = 1, …, *m*_*i*_, is given by Eq (1) [26]. The Cox model was applied to determine the significant covariate, which are associates with LOS The model was fitted using survival package in R [27,28].

$$h\left(t_{i},X_{i}\right)=h_{0}\left(t_{i}\right)\text{exp}(\sum_{j=1}^{p}X_{ij}\beta_{j})$$ (1)

*h*_0_(*t*_*i*_)is the baseline function.

*X*_*i*_(*X*_1*j*_, *X*_2*j*_, …, *X*_*pj*_) is the set of feature n vectors.

### Experiments

The experiments of FS-opa with the mental dataset are displayed in this section. In *Fs-opa* with the raw-full dataset, part presents the main results of *FS-opa* from the raw-full dataset. In addition, in the section of column-constrained datasets illustrate how column-constraint datasets are obtainable by revisiting the dataset armed with simple hypotheses to verifying the consistency of the found results.

#### Fs-opa with raw-full dataset

*FS-opa* is first applied to the dataset as found, without any pre-processing or intervention. The idea is to verify what is possible to model with no previous knowledge of the problem domain. The dataset was obtained from the mental health care information system responsible for coordination of hospitalizations in mental health specialized hospitals in the region of Ribeirao Preto, Brazil and it was composed by 8,755 samples (rows) with 52 features (columns), labeled as shown S1 Appendix. A simple inspection of the dataset shows that the majority of them are male, single and living alone, with white skin color. Patients have aged from 5 to 89 years, among other aspects. Moreover, missing values and some inconsistencies can also be found in the raw dataset. Such information is not used in the modeling through *FS-opa* in the study based on the raw-full dataset, as presented in the sequel.

**Fig 10:**
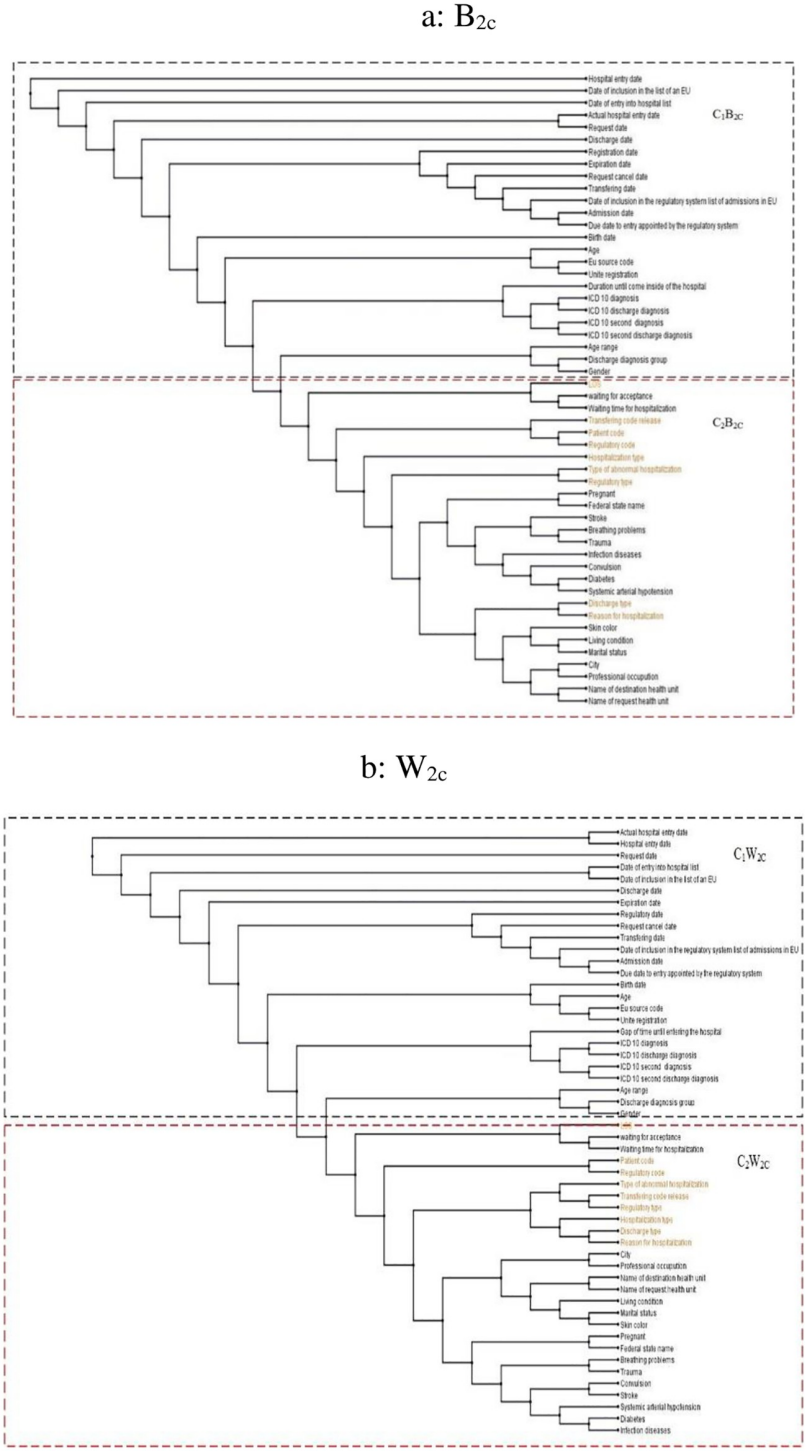
SM1 of B_2c_ and W_2c_. a: B_2c_, b: W_2c_.

**Fig 11:**
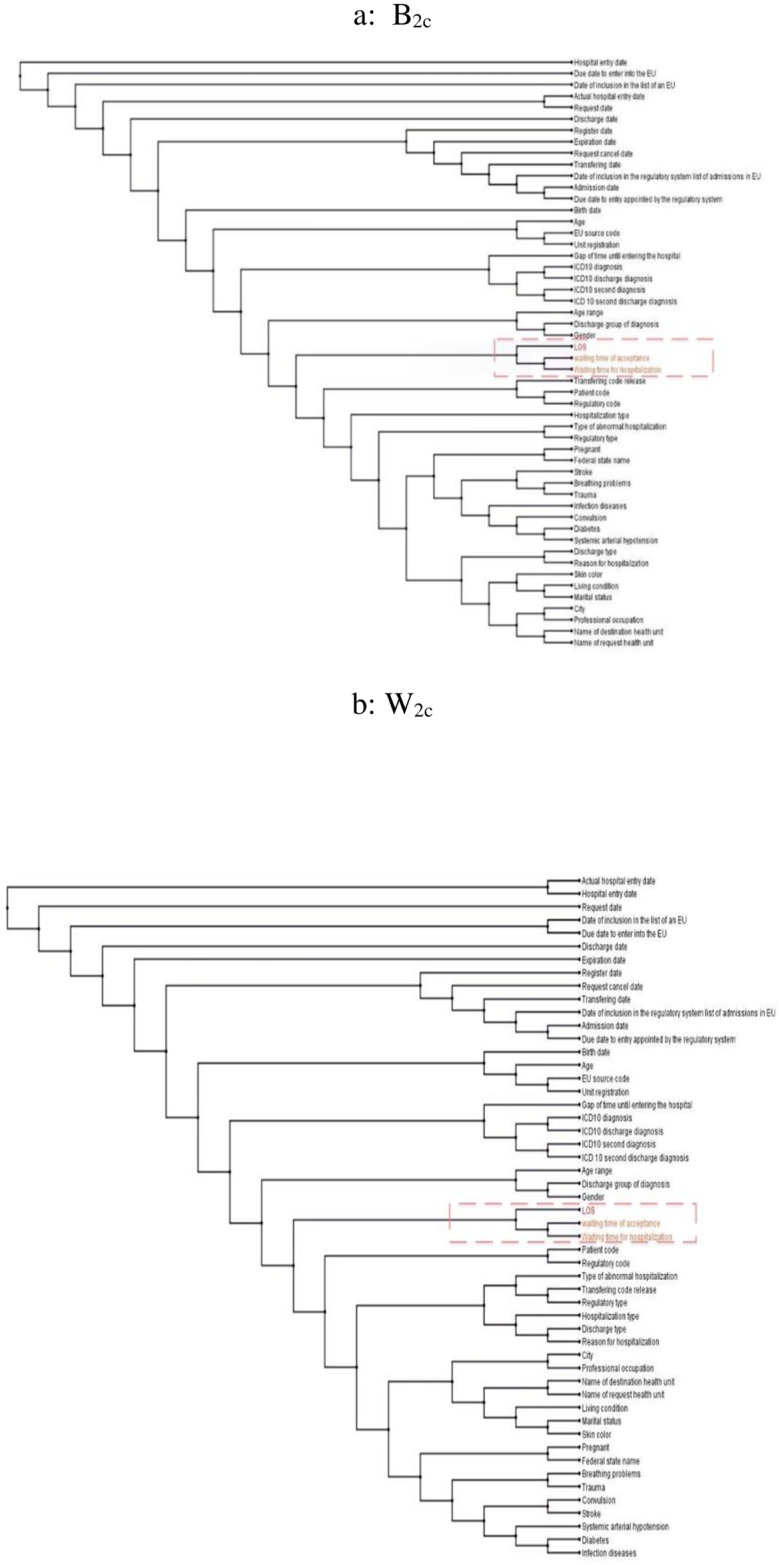
SM2 of B_2c_ and W_2c_. a: B_2c_, b: W_2c_.

It is worth to remark that *FS-opa* first ranks samples according to a criterion and splits the ranks into some categories (2*C*, 4*C* and 8*C*), generating six subsets (*B*_2*C*_, *W*_2*C*_, *B*_4*C*_, *W*_4*C*_, *B*_8*C*_, *W*_8*C*_). Then data associated to each feature (column) is saved into the same file, with the same name of the corresponding column, in a directory with the same name of the subset. Then, FS-opa runs for each directory producing six phylogram-based models. It is worth to remark that *FS-opa* first ranks samples according to a criterion and splits the ranks into some Instead of presenting only set r with all the features selected by *FS-opa*. For instance, Figs 1 and 11 shows the partial results found by procedures SM1 and SM2 of the 2C category to illustrate how the practitioner can manipulate FS-opa to deal with a real scenario.

(Fig 10a) shows the phylogram and the selected variables (orange) obtained by SM1 applied to *B*_2*C*_. Similarly, (Fig 10b) shows the phylogram and the selected variables (orange) produced by SM1 executed for *W*_2*C*_ (Fig 11a and 11b) synthesizes the models and other results generated by SM2 run for *B*_2*C*_ and *W*_2*C*_, respectively.

#### Fs-opa with raw-full dataset

Based on the results from the 52-feature model, some improvement can be achieved by revisiting the dataset armed with simple assumptions. The process of revision is also a way of verifying the consistency of results, as when it is obtained by a resampling procedure. Six datasets, with less than 52 features, were derived from the 52-Feature Dataset (52-FD) and *FS-opa* applied to them. As described in the sequel, the resampling doesn’t require any previous knowledge from the domain of mental health.

A first resampling (RS1), the number of columns is constrained based on simple inspection of the amount of missing values for each variable. Columns with more than 85% with empty cells were removed transfer code release, admission date of emergency section, cancel request date, date of entry into emergency list, date of reserve into emergency section and transfer date generating the 46-Feature Dataset (46-FD).

Another resampling (RS2) produced the 37-feature dataset (37-FD) by removing columns that seem to contain mostly repeated information. The first features related to “date” were removed. For example, admission date and register date are usually both related to instant of time that a patient reached the hospital for the first time. Birth date and age is a similar case since age depends on the patient’s birthday. Note that most of the meaningful information related to time is retained by LOS. Features storing codes (patient code, regulation code, and unit source code) were also removed. Data of type “code” can be seen as redundant labels or IDs. However, they can benefit human or computer-aided management; they are relatively less relevant for modeling than other variables.

Differently from raw-full dataset, RS1 and RS2, the resampling called RS3 is based on knowledge extracted from the results of *FS-opa*, but not on knowledge from experts on the area. Phylogram from 52-FD, 46-FS and 37-FD have some clades preserved in all of them. It means that the features associated by them didn’t reveal any relevant information that enabled *FS-opa* to distinguish “good” samples from “bad” samples. Those features, diabetes, stroke, systemic arterial hypotension, trauma, ICD10 diagnosis, ICD10 discharge diagnosis, ICD10 second discharge diagnosis, infection disease, code patient, code regulation, unit code source, convulsion, date entrance, unite registration and breathing problems less relevant for problem modeling, thus, they were removed from RS2 in order to compose RS3, generating the 21-FD that phylogram trees.

Comparisons of sets r (unions of s_clade_ and s_criterion_) obtained from RS1, RS2 and RS3 (columns three, four and five of in Tables 1 and 2) to the set r obtained from 52-FD (column two of Tables 1 and 2) can generate two new lists: *i*) with common features (RS4), that were selected by FS-opa in at least two of the three resamplings (RS1, RS2 and RS3) and they were also selected from 52-FD; *ii*) with novelty features (RS5), that selected by *FS-opa* in at least two of the three resamplings but they were not selected from 52-FD.

**Table 1:** Clade-based selected lists, s_clade_, obtained by SM1 from 52, 46, 37, and 21-FDs, respectively, Full, RS1, RS2 and RS3 resamplings; as well as, common (9-FD) and uncommon features (6-FD) and union of both sets (15-FD) corresponding to RS4, RS5 and R.

	raw-Full dataset and basic resamplings				Common features in relation to Full	Novelty features in relation to Full	Common and novelty features together
#	52-FD	46-FD (RS1)	37-FD (RS2)	21-FD (RS3)	11-FD (RS4)	6-FD (RS5)	15-FDRS6
1		Age	Age	Age		Age	Age
2	Age range						
3	Code patient	Code patient					
4	Code Regulatory	Code Regulatory					
5	Date birth (*)						
6	Discharge diagnosis group	Discharge diagnosis group	Discharge diagnosis group		Discharge diagnosis group		Discharge diagnosis group
7	Type of discharge	Type of discharge	Type of discharge	Type of discharge	Type of discharge		Type of discharge
8	Hospitalization Type	Hospitalization Type	Hospitalization Type	Hospitalization Type	Hospitalization Type		Hospitalization Type
9	Living condition	Living condition		Living condition	Living condition		Living condition
10	LOS	LOS	LOS	LOS	LOS		LOS
11	Marital status						
12		Pregnancy	Pregnancy	Pregnancy		Pregnancy	Pregnancy
13	Gender	Gender	Gender	Gender	Gender		Gender
14	Skin color						
15	Type of abnormal hospitalization	Type of abnormal hospitalization	Type of abnormal hospitalization	Type of abnormal hospitalization	Type of abnormal hospitalization		Type of abnormal hospitalization
16	Regulatory type	Regulatory type	Regulatory type	Regulatory type	Regulatory type		Regulatory type
17	Reason for hospitalization	Reason for hospitalization		Reason for hospitalization	Reason for hospitalization		Reason for hospitalization
18				Federal State name		Federal State name	Federal State name
19				Waiting time for hospitalization		Waiting time for hospitalization	Waiting time for hospitalization
20				Waiting time of acceptance		Waiting time of acceptance	Waiting time of acceptance

(*) Date birth wasn’t used in Cox model since it has date format.

**Table 2:** Criterion-based selected lists, s_criterion_, obtained by SM2 from 52, 46, 37, and 21-FDs, respectively, Full, RS1, RS2 and RS3 resamplings; as well as, common (9-FD) and uncommon features (6-FD) and union of both sets (15-FD) corresponding to RS4, RS.

	raw-Full dataset and basic resamplings				Common features in relation to Full	Novelty features in relation to Full	Common and novelty features together
#	52-FD	46-FD (RS1)	37-FD (RS2)	21-FD (RS3)	11-FD (RS4)	6-FD (RS5)	15-FD RS6
1				Age		Age	Age
2	Discharge diagnosis group	Discharge diagnosis group	Discharge diagnosis group		Discharge diagnosis group		Discharge diagnosis group
3	LOS	LOS	LOS	LOS	LOS		LOS
4	Gender	Gender	Gender	Gender	Gender		Gender
5	Regulatory type	Regulatory type	Regulatory type		Regulatory type		Regulatory type
6	Waiting time for hospitalization	Waiting time for hospitalization	Waiting time for hospitalization	Waiting time for hospitalization	Waiting time for hospitalization		Waiting time for hospitalization
7	Waiting time of acceptance	Waiting time of acceptance	Waiting time of acceptance	Waiting time of acceptance	Waiting time of acceptance		Waiting time of acceptance
8				Gap of time until entering the hospital		Gap of time until entering the hospital	Gap of time until entering the hospital

Finally, the list with common and novelty features are then combined into RS6. Common features are one estimate of the most robust subset among all the sets *r* that were found. On the other hand, novelty features are features that require resampling to become salient, but with potential possess relevant information for modeling.

A certain concerning is that novelty features can be just noise. Once again, resampling by the bootstrap technique [29] is a way to verify it, but in this thesis, we evaluate it by checking if AIC of the associated Cox model is improved. Note that other information criterion to determine a model representativeness of data can be investigated as, for example, Bayesian Information Criterion (BIC) [30], likelihood ratio test [31], Bayes Factor [32], and minimum description Length [33] as a measure of parsimony.

The lists of features from *s*_*clade*_ and *s*_*criterion*_ obtained by SM1 and SM2 of *FS-opa* from all the seven datasets investigated (52-FD / Full, 46-FS / RS1, 37-FD / RS2, 21-FD / RS3, 11-FS / RS4, 6-FD / RS5, and 15-FD / RS6 are synthesized Tables 1 and 2, respectively.

## Results and discussions

The final step of *FS-opa* (Fig 9) is the construction of Cox model based on the selected lists in order to find the best set.

First Cox regression is applied to each of the seven lists of features investigated. The covariates of Cox model are shown in Tables 1 and 2. The best-fitted model, as well as the significant covariates, can be chosen by using the Akaike Information Criterion (AIC) [34] since we don’t have nested models (two models are nested if one model contains all the terms of the other, and at least one additional term) [35] When we simultaneously analyze the significance of the covariates in the modelling. In order to choose the significant covariates, we proposed to use the forward approach [36] combined with the AIC. Using this method, we keep the covariate in the model if it decreases the AIC value, otherwise it is assumed as non-significant for explaining the target variable, in our case, the LOS in psychiatric hospitals.

**Fig 12:**
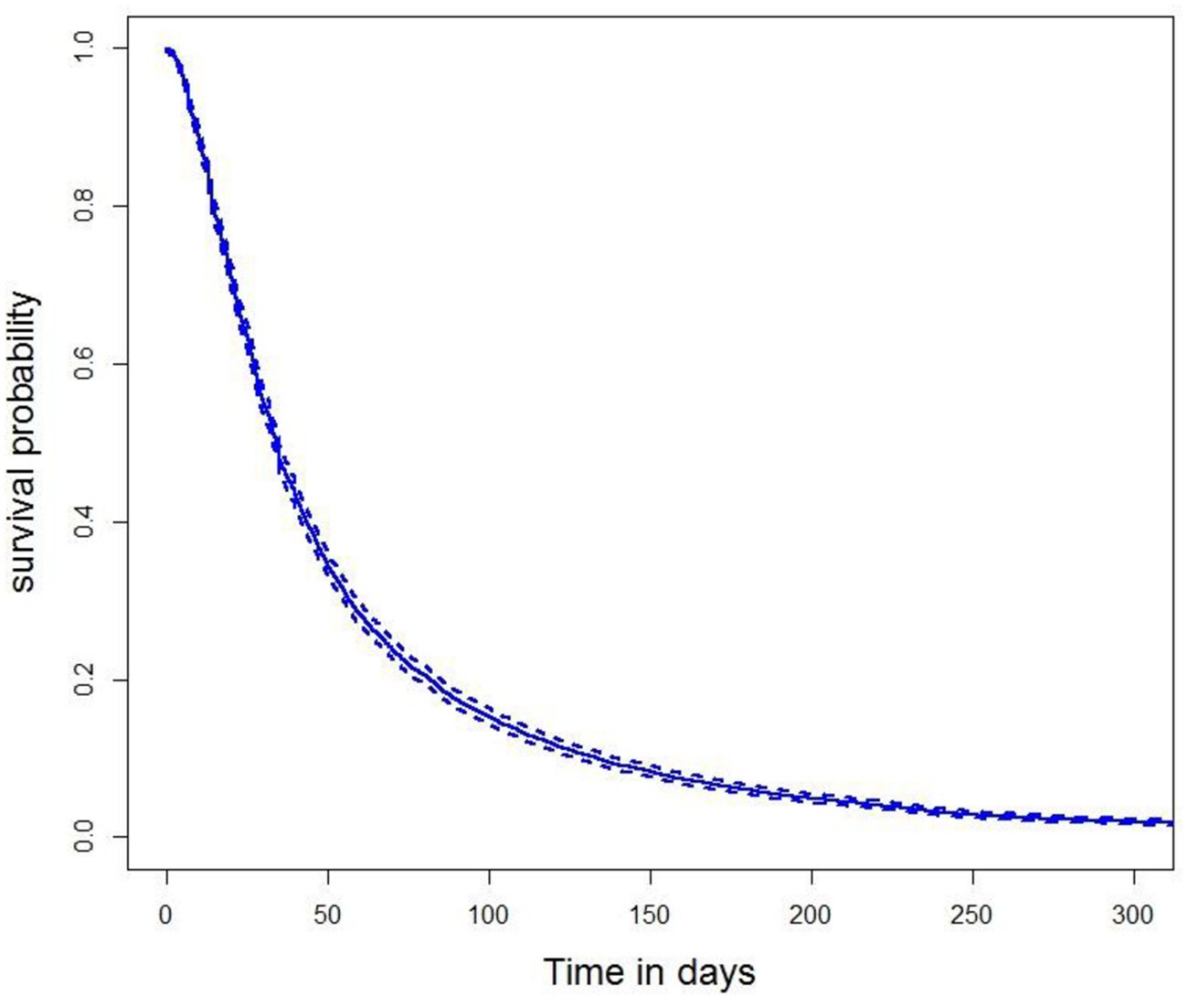
Survival probability according to LOS obtained by the model with best AIC (RS6).

**Fig 13:**
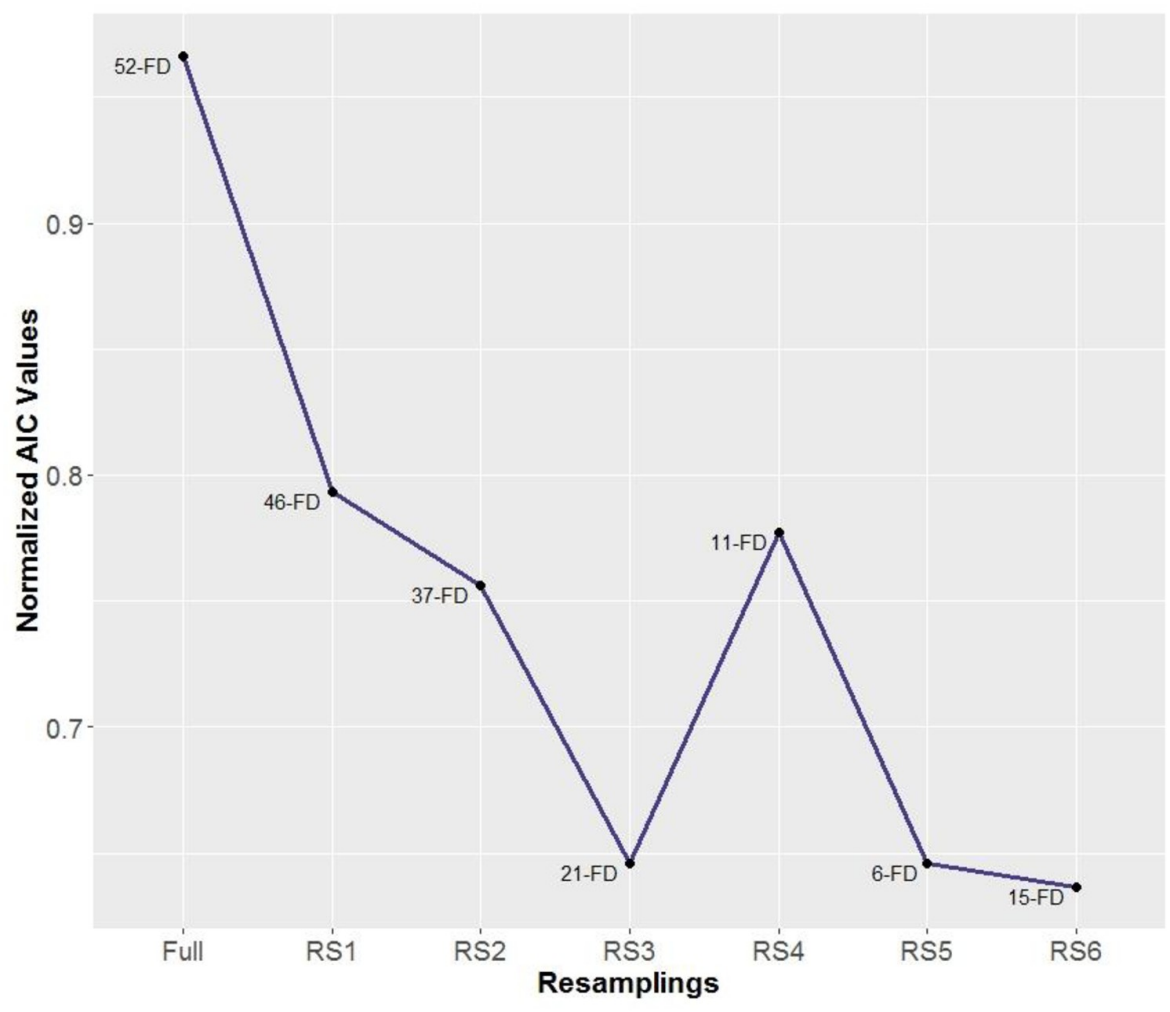
Comparison of the seven Cox models generated according to AIC.

**Fig 14:**
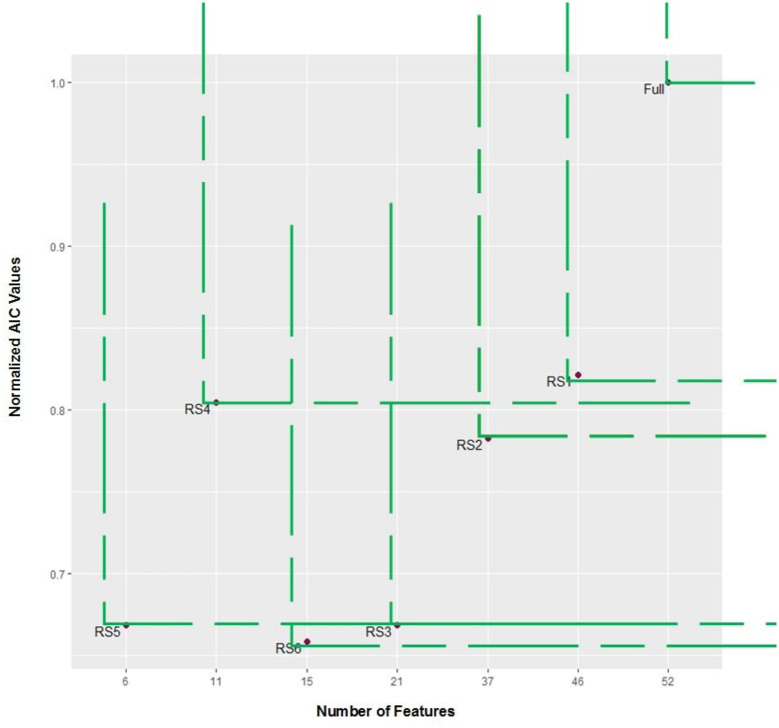
Evaluation of the seven models by a multicriteria decision-making technique. The colored quadrant (rectangle corners) highlights regions dominated by each point (model). RS5 and RS6 are the non-dominated models.

The AIC values related to Cox models obtained are depicted in (Fig 12). The lowest valued for AIC is 68010.27 for the 15-FD based model. It corresponds to RS6, the union of features from RS4 (common features, robust ones) and RS5 (novelty features), thus, such combination seems to be capable of extracting information from data better than other resamplings for modeling through Cox approach.

(Fig 13) presents an evaluation of Cox models according to a multicriteria decision making technique based on non-dominated sets [37] as used by *OPA*. First solutions are plotted in the bi-objective space with the two dimensions Number of Features and Normalized AIC values (normalized with high AIC value = 106868.6). A “rectangle” is associated in the quadrant at the “northeast” of each point (reference). Note that any other point in such a rectangle has equal or highest AIC as well as equal or higher number of features than the reference. We say that a reference point is non-dominated by the points inside its rectangle, in the same way that the points of the rectangle are dominated by the reference point. Thus, the multi-criteria decision making can be applied to the Cox models generated using the seven lists of features in order to compare them.

(Fig 14) shows that RS2 point dominates RS1 point, RS3 point dominates RS2, RS4 dominates RS1, RS6 dominates RS4, RS3, RS2 and RS1, and RS5 dominates RS4, RS2 and RS1. However, no point dominates RS5 and RS6, then both points are non-dominated and compose the best set of models according to multicriteria decision making based on AIC and number of feature objectives. The trade-off between RS5 and RS6 models are clear since RS6 has the lowest AIC (69020.64, while RS6 has AIC equal to 68010.27) but RS5 uses 6 features while RS6 requires 15 features.

**Fig 15:**
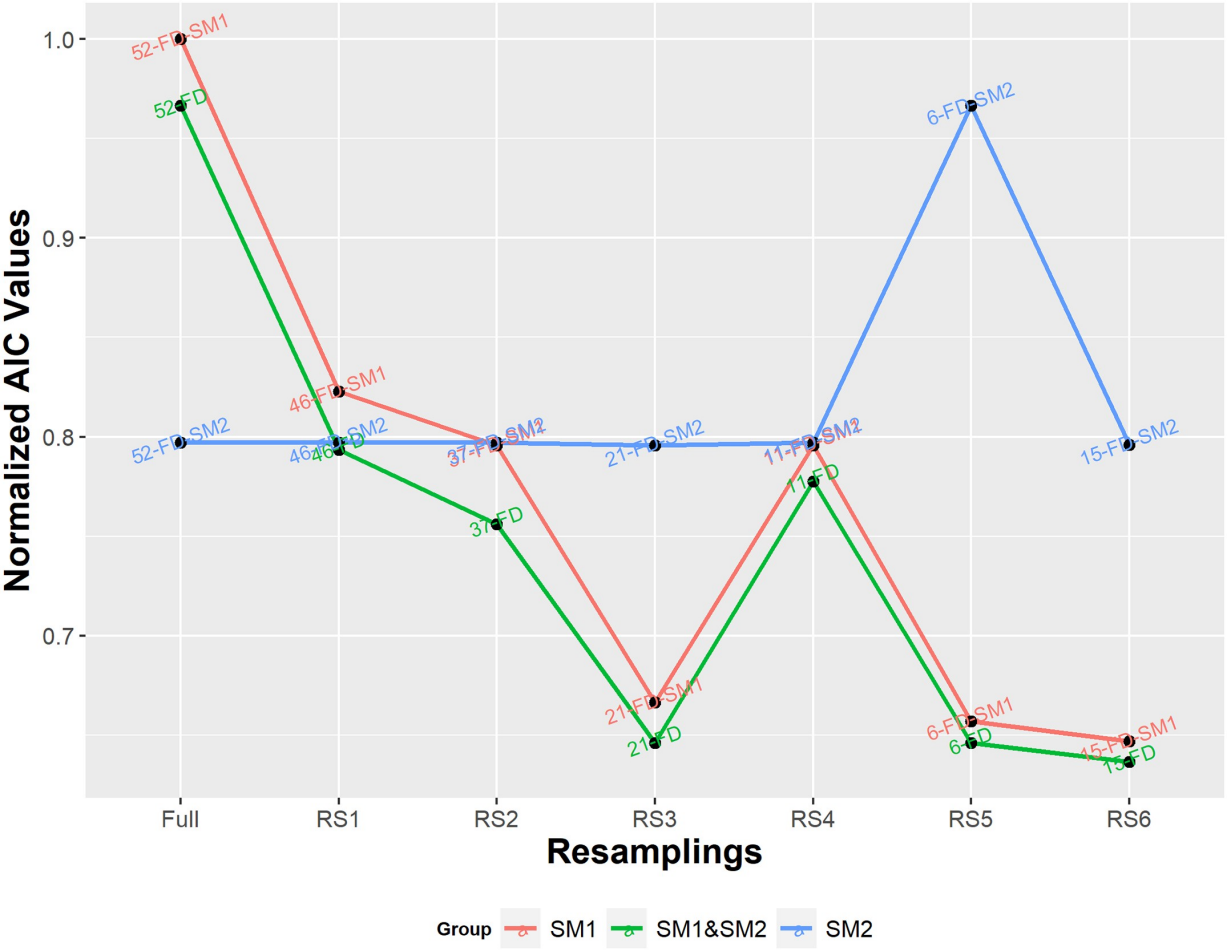
Individual and relative contribution of each dataset to the performance of Cox models.

Next, we compare each of the performance of Cox models for each column-constrained dataset due to the feature lists obtained by each of the procedures SM1 and SM2, as well as those obtained by the complete *FS-opa* (SM1+SM2). (Fig 15) enables the evaluation of the relative contribution of each subset in order to improve Cox models. Clearly, SM1 sensitively provide the largest improvements in AIC, while SM2 increments such performance. Note that SM1 requires no previous knowledge from data, it is data agnostic. On the other hand, SM2 requires at least a target variable, assumed highlighted related to the scope or purpose of the model.

**Fig 16:**
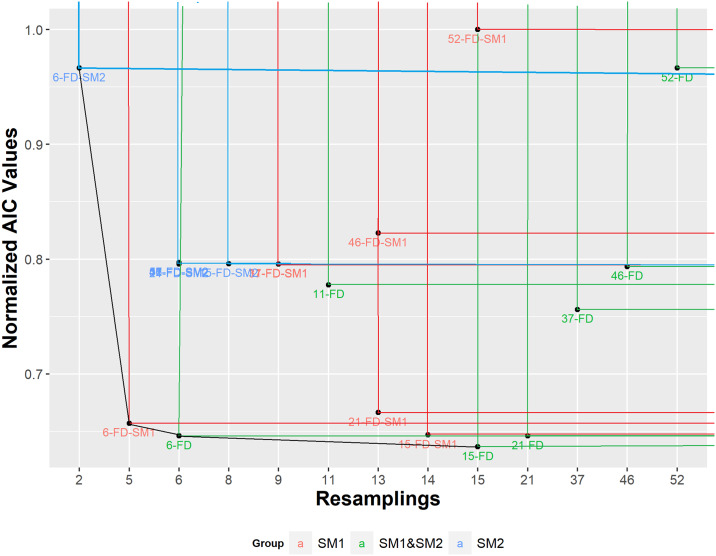
Relative contribution of each set to the Cox models in a trade-off analysis based on non-dominance, according to two criteria: Normalized AIC and number of features used.

In a certain way, the results show that much of the modeling improvements modeling based on the proposed feature sensitivity analysis doesn’t depend on any previous domain knowledge. Such a result is coherent with usual *OPA* achievements since it works with black-box problems (the term related to no knowledge from the problem in the optimization field) and it has found optimal solutions for large-scale multimodal optimization problems.

(Fig 16) presents other relationships among the quality of the Cox models generated according to a bicriteria evaluation, synthesized by the non-dominated sets. A curve or line connecting them in the biobjective space is called a non-dominated front. Beside AIC value, the number of features of each set is another criterion for minimizing, since it is a measure of parsimony. Analysis of trade-offs produces extreme points in the front, corresponding to an adequate value for one criterion and poor value for the other. The Cox model based on 6-FD-SM2 uses the lowest number of variables, while its AIC (larger than 0.95) is near to the largest found, related to 52-FS-SM1 (1.00). On the other hand, the Cox model from 15-FD (SM1+SM2) in the front has 15 variables and the found lowest AIC.

There are several techniques to choose a solution (a set) from a front, but a relatively simple and useful indicates the solution in the elbows of the front. (Fig 16) shows a unique elbow composed by sets 6-FS-SM21 and 6-FD. Both are near to the ideal point (that corresponds to zero AIC and one variable used). According to the number of variables both models are similar, but the 6-FD based model has the second-best found AIC, highlighting it in some way.

Such result was the best *FS-opa* that provided without additional knowledge for the mental health dataset. The evaluation of the relevance of the 6-FD set according to experts on the mental disorder as well as the LOS estimates from the corresponding Cox model are both investigations that should be performed. Although their relevance, they are proposed as future work since it demands a relatively long-time cooperation with experts. In a certain way, such perspective conflicts with the main purpose of the *FS-opa*, to extract as much as possible information from a dataset without previous knowledge from the problem domain. It is important to remark that *FS-opa* results also should contribute for further investigations since: i) solutions in front are the best found ones, thus, by using them, experts will only work with consistent sets, with relatively low level of redundancy; ii) *FS-opa* found high quality models using sets with low number of variables, then any new hypothesis (involving new combinations of those variables or parameter setups of Cox Model) could be tested; and iii) other hypotheses or models based on knowledge from experts can be compared to models in the front, as reference (with no bias and agnostic to data) for evaluating and/or normalizing the improvement of them.

**Fig 17:**
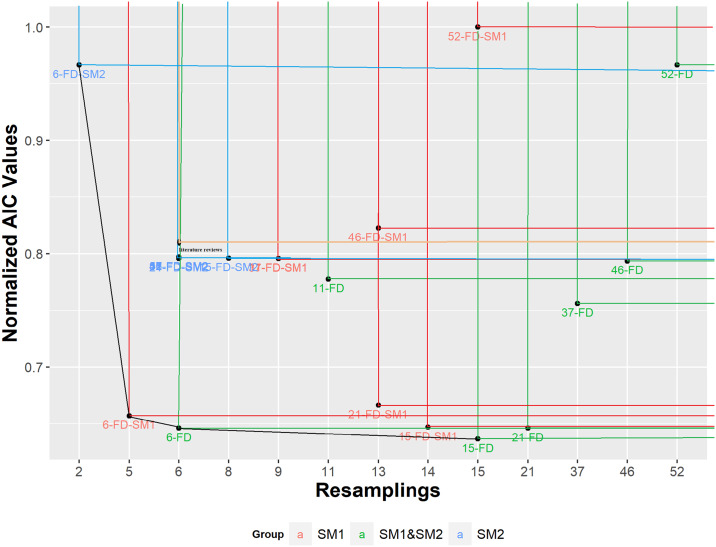
Relative contribution of each set to the Cox models in a trade-off analysis based on non-dominance, according to two criteria: Normalized AIC and number of features used in comparisons with literature reviews.

Finally, (Fig 17) presents another comparison based on bicriteria evaluation of the Cox models generated, but now in relation to the literature results from the literature for mental health disorders. A review of papers in this field found a consensus of the principal features that have been used as indicators for LOS, such as age, gender, living condition, skin color, marital status, and professional occupation. Such a consensus set corresponds to the best model that *FS-opa* generated without additional knowledge.

## Conclusions

The essence of data mining techniques is the possibility of discovering valuable data; they have recently become a predominant field of research with broad applications, specifically in medical healthcare. This study focused on developing a mining approach based on the optimization method called *OPA*, since can work with relatively complex problems without previous knowledge. This method constructs probabilistic models of correlated variables based on hierarchical clustering techniques. *DAMICORE* is one of the main clustering methods employed by *OPA* that was also used in this project. Based on a combination of three other methods (normalized compression distance, Neighbor-Joining and Fast Newman [14] it can deal with different types of dataset together without the previous transformations that may require knowledge from the problem domain.

The main contribution of this study is a new approach for Feature Sensitivity analysis from *OPA*, called *FS-opa*. It also involves the combination of other statistical methods in order to enable practical results from such analysis. For the study case of mental health disorders, the Cox approach for survival analysis was employed for predicting LOS.

Need to mention that the Current feature selection method will require at least a data normalization or rescaling (for example, transform data to real numbers or Binary values) and some treatment-related to the imputation of missing data. That requires some prior knowledge from the domain, and the obtained result is not raw data. Thus, the required comparison is only possible with a violation of our purpose (work directly with raw data without any prior knowledge from the domain). The only way to proceed with the comparison would be in case the raw data has no issues to treat (requiring no expertise form the problem domain). Still, this case is trivial and is not motivated for a more robust data mining method.

Moreover, *FS-opa* can work with a raw dataset, showing that it requires no knowledge from the problem domain to obtain preliminary prediction models. Moreover, the improvement of them from the raw dataset is viable through a series of simple hypothesis among data quality, i.e. that usually doesn’t require knowledge from experts and are checkable by the *FS-opa* together with a multicriteria decision-making approach based on non-dominated sets. In fact, the improvement of data quality is arranged as a resampling procedure, where each new resampling set is determined according to the hypothesis. Results show that the consensus of variables selected from each resampling as well as the novel found features in each resampling are relevant for modeling.

In the experiments performed, the multicriteria decision-making strategy of *FS-opa* to find the best Cox models generated from the resampling procedure found that RS5- and RS6-based models are the non-dominated Cox models with an adequate trade-off. These results also emphasize that it is possible to construct relevant models from a relatively complex raw dataset without prior knowledge from the problem domain.

It also worth to note that our results are consistent with several studies [38–41] Some previous research showed that living with family and not married affected the long duration of psychiatric hospitalization [42,43].

This study demonstrates that the factors related to the time and type of hospitalization in our sample do significantly influence on the LOS. Furthermore, many studies find an association between LOS and diagnosis [41,44,45] We found the diagnosis, treatment type (involuntary, voluntary and compulsory treatment) have a positive impact on the LOS.

Naturally, knowledge from experts must be used when they are available. In this way, FS-opa can be oriented according to it. For example, by using the relative importance of features through the SM2 procedure of *FS-opa*, new criterion-based selected lists can be generated. Then, they can be analyzed together with the other lists obtained by *FS-opa* as in the resampling procedure combined with the multicriteria decision-making approach. Moreover, these findings have important implications for efficient economic management and reduce LOS of psychiatric patients in the health care system. Indeed, the proposed approach enables researchers with little knowledge of the evolutionary computation field to apply *FS-opa* for their dataset.

Our findings improve in some way the psychiatric service and the socioeconomic status of the psychiatry department. Moreover, it can benefit the directions of future studies crucially needed in this area. Due to the increasingly more effective and efficient data collection and storage mechanisms in a variety of medical fields coupled with the enormity of ever more complex problems, *FS-opa* seems a method that can contribute to deal with such complexity. Finally, other approaches based on *FS-opa* principles may enable the improvement of analysis in the areas of healthcare.

## Supporting information

S1 Appendix List of all variables form 52-FD, their meaning in English, the corresponding data types and value ranges. (DOCX)
