# Supplementary material for: Feature sensitivity criterion-based sampling strategy from the Optimization based on Phylogram Analysis (Fs-OPA) and Cox regression applied to mental disorder datasets
Source: PLoS One. 2020 Jul 1;15(7):e0235147. doi: 10.1371/journal.pone.0235147 (PMC7329087; doi:10.1371/journal.pone.0235147)
Supplement: S1 Appendix — (DOCX) [file pone.0235147.s001.docx]

**S1 Appendix. List of all variables form 52-FD, their meaning in English, the corresponding data types and value ranges.**

| **Number** | **Portuguese** | **Meaning in English** | **Type of values / Value range** |
| --- | --- | --- | --- |
| **1** | Idade | Age | Numeric / 0-100 |
| **2** | Faixa_etaria | Age ranges | Numeric / 0-5, 6-18, 19-60, 61-80, 81-100 |
| **3** | Data_nascimento | Birth date | Date / DB period* |
| **4** | Prob_respiratorios | Breathing problems | Nominal / Yes, No,  Empty |
| **5** | Municipio | City | Nominal / City Name |
| **6** | Cod_paciente | Patient code | Numeric / four Arabic digits |
| **7** | Cod_regulacao | Regulatory code | Alphanumeric / F00-F16000 |
| **8** | Cod_unidade_origem | EU** source code | Numeric / 1-199 |
| **9** | Convulsao | Convulsion | Nominal / Yes, No, Empty |
| **10** | Data_cancel_solicitacao | Request cancel date | Date / DB period |
| **11** | Data_lista_reg | Date of inclusion in the regulatory system list of admissions in EUs | Date / DB period |
| **12** | Data_lista_hospital | Date of inclusion in the list of an EU | Date / DB period |
| **13** | Data_internacao | Hospital entry date | Date / DB period |
| **14** | Data_vaga_reg | Due date to entry appointed by the regulatory system | Date / DB period |
| **15** | Data_cadastro | Registration date | Date / DB period |
| **16** | Data_internacao_real | Actual hospital entry date | Date / DB period |
| **17** | Data_admissao_reg | Date when the regulatory system chose an EU | Date / DB period |
| **18** | Data_vaga_hospital | Due date to enter into the EU | Date / DB period |
| **20** | Diabetes | Diabetes | Nominal / Yes, No, Empty |
| **21** | Data_alta | Discharge date | Date / DB period |
| **22** | Grupo_diagnostico_alta | Discharge diagnosis group | Nominal  / (AD, TM, Empty) |
| **23** | Diff _vaga_internacao | Gap of time until entering the Hospital | Numeric / 0-26 days |
| **24** | Data_expiracao | Expiration date | Date / DB period |
| **25** | Tipo_internacao | Hospitalization type | Nominal / Voluntary, Involuntary, Compulsory |
| **27** | Cid10_diagnostico | ICD10 diagnosis | Nominal / E32.3-Z91.5 |
| **26** | Cid10_diagnostico_alta | ICD10 discharge diagnosis | Nominal /A00 - Z73.1 |
| **29** | Cid10_seg_diagnostico | ICD10 second diagnosis | Nominal / B23.8-Z91.5 |
| **28** | Cid10_seg_diagnostico_alta | ICD10 second discharge diagnosis | Nominal / A00-Z32.1 |
| **30** | Doenca_infecto | Infection diseases | Nominal / Yes, No,  Empty |
| **31** | Tempo_internacao | Length of Stay in Hospital (LOS) | Numeric / 0-1521 days |
| **32** | Arranjo_domiciliar | Living Condition | Nominal / Alone, Father, Mother Friend, Other condition |
| **33** | Estado_civil | Marital status | Nominal / Single, Married,  Divorced |
| **34** | Nome_unidade_destino | Name of destination health unit | Nominal / Organization Name |
| **35** | Nome_unidade_solicitacao | Name of the requested health unit | Nominal / Organization Name |
| **36** | Gravidez | Pregnant | Nominal / Yes, No, Empty |
| **37** | Profissao_ocupacao | Professional occupation | Nominal / Employed, Unemployed, Retired |
| **38** | Tipo_regulacao | Regulatory type | Nominal / Yes, No, Empty |
| **39** | Data_solicitacao | Request date | Date / DB period |
| **40** | Sexo | Gender | Nominal / Male, Female |
| **41** | Etnia | Skin color | Nominal / White, Black, Brown, Yellow |
| **42** | UF | Federal state name | Nominal / Two-letter acronym |
| **43** | AVC | Stroke | Nominal / Yes, No, Empty |
| **44** | HAS | Systemic arterial hypotension | Nominal / Yes, No, Empty |
| **45** | Cod_transf_alta | Transferring code release | Numeric |
| **19** | Data transferencia | Transferring date | Date /  DB period |
| **46** | Traumatismo | Trauma | Nominal / Yes, No, Empty |
| **47** | Tipo_alta | Type of discharge | Nominal / Clinic, Transfer, Transfer to mental section, By request, Administrative, Evasion, Died |
| **48** | Tipo_internacao_de | Type of abnormal hospitalization | Nominal / Spontaneous demand,  Transfer from another clinic,  Judicial order, Empty |
| **49** | Unidade_cadastro | unit registration | Numeric / 4-199 |
| **50** | Tempo_espera_internacao | Waiting time for hospitalization | Numeric / 0-365 days |
| **51** | Tempo_espera_aceite | Waiting time for acceptance | Numeric / 0-365 days |
| **52** | Via_internacao | Reasons for hospitalization | Nominal / Spontaneous Demand, Caregiver, EU |

DB Period - period of data collected available in the database used, from 7/12/2012 to 12/30/2017

EU - Emergency Unit
